# Extended recovery of cardiac function after severe infantile cardiomyopathy presentation of Barth syndrome

**DOI:** 10.1002/jmd2.12264

**Published:** 2021-12-28

**Authors:** Jessie Yester, Brian Feingold

**Affiliations:** ^1^ Heart Institute UPMC Children's Hospital of Pittsburgh Pittsburgh Pennsylvania USA; ^2^ Department of Pediatrics University of Pittsburgh School of Medicine Pittsburgh Pennsylvania USA; ^3^ Clinical and Translational Science University of Pittsburgh School of Medicine Pittsburgh Pennsylvania USA

**Keywords:** Barth syndrome, cardiomyopathy, heart failure, noncompaction, pediatric

## Abstract

Cardiomyopathy is the most common presenting feature of Barth syndrome, often presenting in infancy with severe heart failure and cardiac dysfunction. Historically, affected infants commonly died early after presentation, sometimes before a diagnosis of Barth syndrome was made. With increases in awareness of Barth syndrome and in the care of infants with severe heart failure, survival of children with Barth syndrome and severe heart failure has improved. We describe our experience caring for five unrelated boys with Barth syndrome who presented with severe cardiomyopathy and heart failure prior to age 2 who have had marked improvement with long‐term response to medical heart failure therapy.

**Synopsis**

Early recognition and appropriate medical therapy can lead to sustained resolution of symptomatic heart failure and improvement in the cardiomyopathy phenotype of Barth syndrome.

## 1 INTRODUCTION

Barth syndrome is a rare multisystem disorder characterized by cardiomyopathy, skeletal muscle weakness, growth delay, and neutropenia.^1^ Pathogenic variants of *TAFAZZIN* on chromosome Xq28.12 result in abnormal cardiolipin metabolism and mitochondrial dysfunction.^2^, ^3^ Although first reported in 1983, relatively few children have been diagnosed, with just over 300 unrelated cases known to the Barth Syndrome Foundation genetic database (http://www.barthsyndrome.org/).

Cardiomyopathy is the most common presenting feature.^4^, ^5^ Although late childhood onset cardiomyopathy has been reported,^6^ more commonly Barth syndrome presents in infancy with severe cardiac dysfunction.^7^, ^8^, ^9^ The phenotype of cardiomyopathy in Barth syndrome is typically either dilated, noncompacted, or a mixture of both.^2^ There is also thought to be an increased risk of life‐threatening arrhythmias in Barth syndrome,^10^ although it is unclear if this risk relates to the degree of cardiac dysfunction or is inherent to the condition alone. Historically, affected individuals commonly succumbed to cardiac death shortly after presentation, sometimes before a definitive diagnosis was made.^8^, ^11^, ^12^ In the more recent era, survival for children with heart failure due to cardiomyopathy has improved,^13^ and it has been our experience that children with Barth syndrome and early onset severe heart failure have also benefited. Herein we describe our experience caring for four unrelated boys with Barth syndrome who presented with severe cardiomyopathy and heart failure in infancy and have had extended resolution of left ventricular (LV) dilation with normalization of LV systolic function (i.e., reverse remodeling) with medical heart failure management. We also report on one additional individual that presented in infancy with heart failure and dilated cardiomyopathy (DCM) who had extended improvement in heart failure symptoms with reverse remodeling for 12 years on heart failure treatment before he ultimately required transplantation for restrictive cardiomyopathy with preserved systolic function.

## 2 CASE SERIES

**FIGURE 1:**
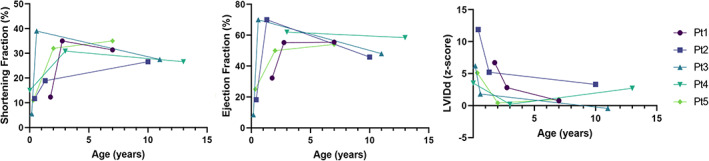
Longitudinal changes in echocardiographic shortening fraction, ejection fraction, and left ventricular internal dimension in diastole (LVIDd) z‐score for all five patients

A summary of select clinical, echocardiographic, and ECG features is shown in Table 1. Graphical summary of longitudinal changes in shortening fraction, ejection fraction, and left ventricular internal dimension in diastole (LVIDd) z‐score on echocardiogram are shown in Figure 1.

**TABLE 1:** Longitudinal cardiac and clinical features

Presentation															
	Age	Weight (kg)	Weight (%tile)	Height (cm)	Height (z‐score)	EF (%)	LVIDd (cm)	LVIDd (z‐score)	LVIDs (cm)	LVIDs (z‐score)	PR (ms)	QRS (ms)	QT/QTc (ms)	QRS axis (°)	Discharge filgrastim
Pt1	21 months	9.71	**−2.2**	83.3	−0.6	**32**	4.4	**6.7**	3.8	**11.4**	158	72	334/**478**	84	No
Pt2	6 months	4.6	**−4.2**	66	−0.6	**19**	4.7	**11.9**	4.1	**18**	140	76	282/430	62	No
Pt3	2 months	3.13	**−3.8**	52.0	**−3.8**	**9**	3.1	**6.2**	2.9	**9.9**	112	60	268/443	43	**Yes**
Pt4	2 days	2.6	**−2.2**	52.0	0.8	**29**	2.5	**3.5**	2.1	**7.5**	104	64	348/**527**	53	No
Pt5	4 months	4.65	**−3.3**	60.5	−2.0	**25**	3.5	**6.9**	3.1	**12.5**	112	76	356/**549**	70	No

At first echocardiogram with marked improvement															
	Age	Weight (kg)	Weight (%tile)	Height (cm)	Height (z‐score)	EF (%)	LVIDd (cm)	LVIDd (z‐score)	LVIDs (cm)	LVIDs (z‐score)	PR (ms)	QRS (ms)	QT/QTc (ms)	QRS axis (°)	Using filgrastim
Pt1	38 months	12.25	−1.5	87.0	**−2.4**	55	3.3	1	2.1	1.1	158	64	366/442	60	No
Pt2	21 months	8.24	**−3.8**	71.0	**−4.9**	**38**	3.7	**5.2**	3.0	**8.3**	118	76	326/**456**	50	No
Pt3	8 months	6.31	**−3.3**	61.0	**−4.3**	**45**	2.6	0.7	1.6	0.5	120	66	306/**446**	43	**Yes**
Pt4	26 months	10.3	**−2.2**	82.0	−1.8	**53**	3.1	0.7	2.0	1.6	124	70	314/436	16	**Yes**
Pt5	22 months	11.8	−0.5	79.0	**−2.4**	**49**	2.9	0	2.0	1.1	102	64	240/402	62	No

Most recent															
	Age	Weight (kg)	Weight (%tile)	Height (cm)	Height (z‐score)	EF (%)	LVIDd (cm)	LVIDd (z‐score)	LVIDs (cm)	LVIDs (z‐score)	PR (ms)	QRS (ms)	QT/QTc (ms)	QRS axis (°)	Using filgrastim
Pt1	7 years	18.95	−1.6	114.4	−1.5	56	3.7	0.8	2.6	1.4	156	72	390/449	39	No
Pt2	10 years	23.85	**−2.2**	127.0	**−2.0**	**46**	4.7	**3.3**	3.5	**4.5**	140	74	364/435	44	No
Pt3	11 years	24.95	**−2.7**	134.5	−1.6	**48**	3.8	−0.4	2.7	1.0	138	76	354/438	−7	**Yes**
Pt4	13 years	38.75	−1.1	155.6	−0.4	58	5.2	**2.7**	3.7	**3.2**	138	94	334/401	62	**Yes**
^a^Pt5	13 years	36.05	−1.7	146.0	−1.8	56	4.9	2.0	3.4	**2.5**	176	98	382/481	87	No

Abbreviations: EF, ejection fraction; LVIDd, left ventricular internal diameter in diastole; LVIDs, left ventricular internal diameter in systole; Pt, patient.

^a^ Data at time of heart transplant evaluation. QTc ≥450 ms considered abnormal. Bold font indicates abnormal value.

### 2.1 Patient 1

**FIGURE 2:**
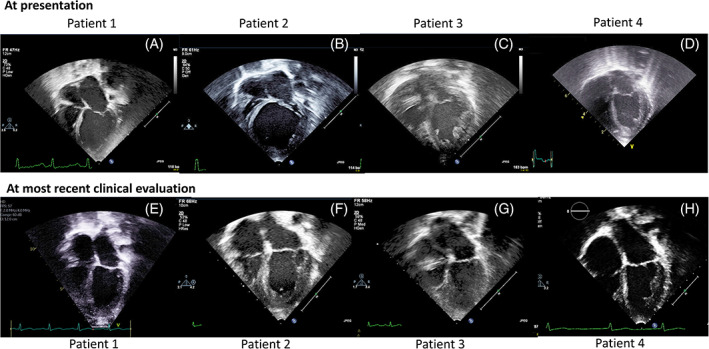
Echocardiogram apical four‐chamber view at presentation (A–D) and latest follow‐up (E–H) for patients 1–4 who have had sustained resolution of heart failure and improvement of cardiac function. Improvement in left ventricular (LV) size is evident in all patients. LV dilation is most evident in (B) and noncompaction most evident in (C)

P1 had a history of hypotonia and undiagnosed developmental delay and presented at age 21 months with acute onset left sided weakness in the setting of a presumed respiratory viral infection. While his facial droop resolved prior to arrival in the emergency room, initial examination was significant for a II/VI blowing systolic murmur at the apex, a gallop, and hepatomegaly. Brain MRI confirmed a right middle cerebral artery stroke in the setting of embolic occlusion of the right internal carotid artery as well as a left parietal stroke. Chest x‐ray demonstrated cardiomegaly with increased pulmonary vascularity. As shown in Figure 2(A), echocardiogram showed severe LV dilation (LV internal diameter in diastole [LVIDd] 4.4 cm, z‐score 6.7; LV internal diameter in systole, LVIDs 3.8 cm, z‐score 11.4) with moderately to severely decreased LV systolic function (shortening fraction [SF] 12%, ejection fraction [EF] 32%). There was also moderate mitral valve insufficiency. The left atrium (LA) was moderately enlarged and there was a 16 mmHg gradient across a stretched patent foramen ovale, consistent with LA hypertension. Echocardiography also showed multiple echogenic LV masses concerning for thrombus, consistent with an embolic carotid artery occlusion and stroke. B‐type natriuretic peptide (BNP) level on admission was 2255 pg/ml (normal <100 pg/ml).

P1 was admitted to the Cardiac Intensive Care Unit (CICU) where he was intubated and started on milrinone and epinephrine to support cardiac output. He was treated with enoxaparin for his thromboembolic stroke. Workup of his DCM revealed 3‐methylglutaconic aciduria and he was ultimately confirmed to have a missense mutation in exon 2 of *TAFAZZIN (c*.*222C > A)*, consistent with Barth syndrome, as well as 15q11.2q13 and 15q13.3 microduplications.

P1 was discharged after a 20‐day inpatient stay. His medical heart failure management at discharge included captopril three times a day (0.9 mg/kg/dose), carvedilol twice a day (0.05 mg/kg/dose, with plans to uptitrate), and furosemide twice a day (1 mg/kg/dose). While he has remained well compensated on medical oral heart failure therapies since initial hospital discharge, his echocardiogram initially began to show subtle improvement in LV dilation and dysfunction by 4 months of therapy, with progressive improvements in LV size and systolic function noted at 7 and 9 months after starting treatment. By 12 months on oral medical heart failure therapies, his LV size was only mildly dilated (LVIDd 3.6 cm, z‐score 2.8; LVIDs 2.3 cm, z‐score 2.6) with a normal EF 55% and by 16 months on therapy (age 38 months) both LV size and EF were normal. He remains on an angiotensin converting enzyme‐inhibitor (ACE‐inhibitor), now enalapril, at last evaluation at age 7 years, having weaned off both furosemide and carvedilol with normal LV size (LVIDd 3.7 cm, z‐score 0.8; LVIDs 2.6 cm, z‐score 1.4; Figure 2(E)) and systolic function (SF 31%, EF 56%) and mildly reduced global longitudinal strain (GLS) of −17.3% (normal −19.5 to −20.8%).

### 2.2 Patient 2

P2 presented to the emergency department at almost 6 months of age for persistently increased work of breathing, wheezing, cough, and cardiomegaly on chest x‐ray. Past medical history was significant for vesicoureteral reflux, asymmetric crying facies, polydactyly, sacral dimple, and poor weight gain. Initial examination revealed no murmur or gallop, but hepatomegaly was appreciated. As shown in Figure 2(B), echocardiography showed a severely dilated left ventricle (LVIDd 4.7 cm, z‐score 11.9; LVIDs 4.1 cm, z‐score 18.0) with severely depressed LV systolic function (SF 12%, EF 19%). He also had prominent LV trabeculations, mild to moderate mitral valve insufficiency, and moderate LA enlargement. BNP level was 2801 pg/ml. He was admitted to the CICU and started on furosemide and captopril. He was initiated on enoxaparin for thromboembolism prophylaxis given his severely decreased LV function. Hematological evaluation revealed neutropenia and he was found to have elevated amounts of urine 3‐methylglutaconic acid. Genetic testing was later consistent with Barth syndrome, showing a predicted pathogenic variant in *TAFAZZIN* (c.778‐24_778‐7delinsA) that had not previously been reported.

P2 was discharged after a 14‐day hospital stay on oral heart failure therapy of captopril three times a day (0.5 mg/kg/dose) plus furosemide every morning (1 mg/kg/dose). After readmission 6 weeks later for worsening heart failure symptoms in the setting of a febrile illness, digoxin twice daily (4.7 mcg/kg/dose) was added. He subsequently was readmitted 3.5 months after starting heart failure therapy for gastrostomy button placement due to poor weight gain. Although echocardiogram at 8 months after diagnosis continued to show severe LV dilation and dysfunction with subtle improvement when normalized for body surface area (LVIDd 4.6 cm, z‐score 5.2; LVIDs 4.1 cm, z‐score 12.6), by 10.5 months on heart failure therapy there was clear improvement in LV size (LVIDd 3.7 cm, z‐score 5.2; LVIDs 3.0 cm, z‐score 8.3) and systolic function (SF 19%, EF 38%). At this point furosemide was weaned and three times daily captopril transitioned to twice‐daily enalapril for convenience in dosing. Further improvement (LVIDd 2.9 cm, z‐score 1.0; LVIDs 2.0 cm, z‐score 1.9; SF 31%, EF 45%) was observed on echocardiogram at 14 months on therapy (age 21 months), though most echocardiograms since have shown mild LV dilation (LVIDd and LVIDs z‐scores 2–3.5) with mildly decreased systolic function (EF 45–50%). Carvedilol was added at age 20 months.

P2 was last seen at age 10 years and had no heart failure symptoms on twice daily enalapril (0.26 mg/kg/dose), carvedilol (0.26 mg/kg/dose), and digoxin (2.6 mcg/kg/dose). His most recent echocardiogram showed a mildly dilated and globular LV left ventricle (LVIDd 4.7 cm, z‐score 3.3; LVIDs 3.5 cm, z‐score 4.5; Figure 2(F)) with prominent trabeculations with mildly decreased systolic function (SF 26%, EF 46%) and strain (GLS ‐19.0%). His BNP was normal at 20 pg/ml. Periodic screening for arrhythmias with ambulatory ECG monitoring has demonstrated no arrhythmias.

### 2.3 Patient 3

P3 presented at 2 months of age for an episode of lassitude and pallor and was found to have cardiomegaly on chest x‐ray. He was initially admitted to the NICU for a sepsis evaluation; however, was transferred to the CICU after echocardiography showed severe LV dilation (LVIDd 3.1 cm, z‐score 6.2; LVIDs 2.9 cm, z‐score 9.9; Figure 2(C)) with severely decreased systolic function (SF 6%, EF 9%). Echocardiogram also showed moderate LV hypertrophy and prominent apical trabeculations, consistent with noncompaction (Figure 2(C)). His BNP was 1940 pg/ml. He was started on milrinone and epinephrine in the cardiac intensive care unit. Laboratory evaluation to elucidate the underlying etiology of his cardiomyopathy was significant for neutropenia but negative for 3‐methylglutaconic aciduria. Barth syndrome was suspected clinically and later confirmed by genetic testing which revealed a pathogenic variant in exon 10 of *TAFAZZIN* (c.710_711del).

P3 was discharged after a 12‐day hospital stay during which he was transitioned to oral heart failure medications. On discharge he was taking aspirin daily (6 mg/kg/dose), captopril three times a day (1.1 mg/kg/dose), digoxin twice a day (3.8 mcg/kg/dose), and furosemide twice daily (1.3 mg/kg/dose). His systolic LV function by echocardiography had improved on discharge (EF 18%), with stable LV dilation. Two months after discharge, his LV was less dilated (LVIDd 2.5 cm, z‐score 1.9; LVIDs 2.0 cm, z‐score 4.0) and systolic function mildly improved (SF 23%). On his subsequent echocardiogram at 6 months on heart failure therapy (at 8 months of age), his LV systolic function was normal (SF 39%, EF 70%), with normal LV size (LVIDd 2.6, z‐score 0.7; LVIDs 1.6, z‐score 0.5). While he remains on chronic ACE‐inhibitor therapy, furosemide was stopped at 10 months and digoxin 13 months after diagnosis. He was last seen in clinic at 11 years of age. As shown in Figure 2(G), his echocardiogram showed normal LV dimensions (LVIDd 3.8 cm, z‐score −0.4; LVIDs 2.7 cm, z‐score 1.0), low normal LV systolic function (SF 28%, EF 48%) and strain (GLS ‐15.3%), and no heart failure symptoms on enalapril twice a day (0.3 mg/kg/dose). His BNP was normal (11 pg/ml). Periodic screening for arrhythmia with ambulatory ECG monitoring has not identified any significant ectopy.

### 2.4 Patient 4

P4 presented on day of life two after becoming tachypneic and hypothermic in the newborn nursery. A chest x‐ray demonstrated cardiomegaly and he was acidotic with lactate of 16 mmol/L. He was resuscitated with intravenous fluids, intubated, and started on intravenous epinephrine and milrinone, and transferred to the CICU. There was a strong family history of infantile death of five maternal great uncles between 1 week and 5 months of birth from heart failure. This raised a clinical suspicion for Barth syndrome that was later confirmed by genetic testing showing a missense pathogenic variant in *TAFAZZIN* (c.347G > A). As shown in Figure 2(D), initial echocardiogram demonstrated a mildly dilated left ventricle (LVIDd 2.5 cm, z‐score 3.5; LVIDs 2.1 cm, z‐score 7.5) with moderate to severely decreased systolic function (SF 15%, EF 29%). He was evaluated and listed for heart transplantation, however, was able to wean gradually off intravenous inotropic and onto oral heart failure therapies. He was inactivated on the transplant list upon hospital discharge at 1 month of age, though his echocardiogram was similar to admission (LVIDd 2.6 cm, z‐score 4.0; LVIDs 2.3 cm, z‐score 6.6; SF 10%, EF 24%). His medical heart failure management at discharge included captopril every 6 hours (0.08 mg/kg/dose), digoxin twice a day (3.2 mcg/kg/dose), furosemide twice a day (1 mg/kg/dose), and aspirin daily (5 mg/kg/dose). He required a brief (2 days) readmission at 6 weeks of age due to lassitude and anemia in the setting of a presumed viral gastroenteritis. His symptoms improved after red blood cell transfusion.

During close outpatient follow‐up, his oral heart failure regimen was augmented and his echocardiogram showed improvement in LV size at 9 months of age (LVIDd 2.9 cm, z‐score 2.0; LVIDs 2.1 cm, z‐score 3.8) with continued decreased systolic function (SF 28%, EF 38%). He continued to gain weight on oral heart failure therapies (enalapril, carvedilol, digoxin, furosemide, and aspirin) with similar echocardiogram findings until 26 months of age when the LV size and systolic function were both essentially normal (LVIDd 3.1 cm, z‐score 0.7; LVIDs 2.1 cm, z‐score 1.6; SF 33%, EF 53%). He was last seen in cardiology clinic at 13 years of age with long‐standing symptoms of easy fatigability, thought to be secondary to peripheral muscle involvement from Barth syndrome, and episodic emesis that has not been responsive to multiple gastroenterology evaluations and medication trials or transition from ACE‐inhibitor to angiotensin receptor blocker. He has no hepatomegaly and BNP levels are consistently normal (last 43 pg/ml). His most recent echocardiogram (Figure 2(H)) showed mild LV dilation (LVIDd 5.2 cm, z‐score 2.7; LVIDs 3.7 cm, z‐score 3.2) with normal systolic function (SF 29%, EF 58%). Although strain assessment was not performed on this study, 7 months prior GLS was −22.7%. His current cardiac medications include losartan daily (1.4 mg/kg/day), carvedilol twice a day (0.26 mg/kg/dose), and digoxin (3.5 mcg/kg/day). On serial ambulatory ECG monitoring performed approximately annually there has been no significant ectopy.

### 2.5 Patient 5

P5 presented at 4.5 months of age with 2–3 months of decreased oral intake and failure to thrive. He was referred for cardiology evaluation due to tachypnea and cardiomegaly on chest x‐ray and on examination had a laterally displaced apical impulse with a subtle gallop but no hepatomegaly. Family history was positive for a maternal uncle with infantile onset cardiomyopathy and heart failure who died at age 4 years. Echocardiogram demonstrated moderate to severe LV dilation (LVIDd 3.5 cm, z‐score 6.9; LVIDs 3.1 cm, z‐score 12.5) with severely decreased function (SF 11%, EF 25%). He was admitted for further evaluation into the etiology of his cardiomyopathy and started on medical heart failure management with captopril and furosemide. Metabolic evaluation was significant for 3‐methylglutaconic aciduria and genetic evaluation initially demonstrated a nonpathogenic *TAFAZZIN* allele (IVS1‐17C > T) in the intron 1 coding region. Clinically he was suspected of having Barth syndrome, and multiple investigations for other etiologies were negative until whole exome sequencing at age 11 years revealed a *TAFAZZIN* pathogenic variant (c.778‐24_778‐7delinsA).

During outpatient follow‐up his echocardiogram remained unchanged at 6, 9, and 12 months on heart failure therapy with ACE‐inhibitor (captopril, later changed to enalapril), carvedilol, and furosemide; however, after 18 months on heart failure treatment (22 months of age) his echocardiogram was much improved (LVIDd 2.9, z‐score 0; LVIDs 2.0, z‐score 1.1; SF 30%, EF 49%). His echocardiogram was stable over the next 5 years until at age 7 years, it began to show atrial dilation and elevated pulmonary artery pressure; features consistent with diastolic dysfunction/restrictive physiology. Over the next 6 years on the same heart failure medications (uptitrated for growth), his echocardiogram showed progressive biatrial dilation, mitral, and tricuspid insufficiency, and pulmonary hypertension with stable, mild LV dilation (LVIDd z score 2–3), and normal to mildly decreased systolic function (EF 47–55%). Around age 12, he began to manifest dyspnea on exertion and cardiac catheterization confirmed elevated filling pressures with elevated, but reactive pulmonary vascular resistance (PVR 5 indexed Wood units). Serial ambulatory ECG monitoring from diagnosis performed approximately annually showed no significant ectopy. In the absence of effective medical therapies for restrictive cardiomyopathy with associated pulmonary hypertension, he was referred for heart transplantation, which he received at age 14 years. He remains well at 1 year after transplantation with resolution of pulmonary hypertension (Figure 2).

## 3 DISCUSSION

In this series, we present five children with Barth syndrome who presented under age 2 with symptomatic heart failure who had extended responses to medical heart failure therapy with resolution of heart failure symptoms and near normalization to normalization of LV size and systolic function. This is important because the prevailing description of outcomes for infants with severe cardiomyopathy and Barth syndrome in the medical literature is commonly of death, need for mechanical circulatory support, and/or heart transplantation.^8^, ^12^, ^14^, ^15^, ^16^, ^17^ During the timeframe that these children were cared for at our center (December 2006–present) we had no other individuals with Barth syndrome die. During his time we also cared for a 3‐year‐old, not reported herein, with Barth syndrome (*TAFAZZIN* c.647G > T) who had decreased cardiac function and medically treated heart failure upon outpatient transfer of care to our center who shortly thereafter required ventricular assist device support and subsequent heart transplantation. While outcomes after heart transplantation for individuals with Barth syndrome were recently shown to be the same as for matched peers with DCM without Barth syndrome,^18^ transplantation has important limitations, most notably inadequate allograft survival leading to death or retransplantation.^19^ Thus, the knowledge that children with Barth syndrome and symptomatic heart failure with severe LV dilation and dysfunction are capable of reverse remodeling and extended recovery is impactful. Specifically, it can inform management decisions about proceeding to transplantation quickly or with a cautious delay while awaiting potential heart failure improvement.

Normalization of LV size and function has been reported to occur in approximately 20% of children with DCM, with younger age and less severe LV dilation at diagnosis reported as predictors in a Pediatric Cardiomyopathy Registry (PCMR) study.^20^ Although the individuals we have reported on had LV dimension z‐scores more consistent with the PCMR non‐recovery cohort, due to small sample sizes it is not appropriate to make predictive comparisons about the capacity of individuals with Barth syndrome to respond to medical heart failure therapy relative to non‐Barth individuals. While highest class of evidence data on effective pediatric heart failure therapies are lacking, it is commonly accepted that ACE‐inhibitors (e.g., captopril, enalapril, lisinopril) or angiotensin receptor blockers (e.g., losartan, valsartan) and beta‐blockers (e.g., carvedilol, metoprolol) form the cornerstone of effective pediatric heart failure therapy for dilated cardiomyopathies of various etiologies.^21^, ^22^, ^23^ More recently, aldosterone receptor antagonists (e.g., spironolactone, eplerenone) have been incorporated into chronic pediatric heart failure medication regimens, and there is anticipation about the potential of valsartan–sacubitril in pediatric heart failure,^24^ which has been shown to be superior to ACE‐inhibitors/angiotensin receptor blockers in adult heart failure with reduced EF.^25^ While anticoagulation to prevent thromboembolic complications is recommended in children with cardiomyopathy and decreased systolic function,^26^, ^27^ aspirin or low‐dose anticoagulation is commonly used for patients with Barth syndrome and noncompaction phenotype with mild to no dysfunction.^28^

The decision to continue indefinite medical HF therapy in these boys deserves brief mention. While all had resolution of their clinical HF symptoms, not all achieved normalization of all echocardiographic parameters and some later showed decline in SF/EF and/or increase in LV dilation. Furthermore, the underlying substrate for HF (i.e., Barth syndrome) remains and individuals with Barth syndrome have been described to have improvement followed by worsening over time. An improved understanding of why cardiac function varies over time is vitally important to optimally caring for individuals with Barth syndrome. In lieu of this, we have elected to maintain all of our patients with Barth syndrome who have not required transplantation on indefinite HF medications. Further, there is randomized clinical trial evidence in adults with nonischemic DCM who had resolution of HF and LV reverse remodeling on medical HF therapy that shows frequent relapse of HF and LV dilation/dysfunction following withdrawal of therapy.^29^

It is important to acknowledge that the improvements in these five individuals were achieved through a combination of awareness of the diagnosis of Barth syndrome, provision of multidisciplinary care (including subspeciality heart failure cardiologists, intensivists, and geneticists), and receipt of appropriate acute and chronic heart failure therapies. Transplant‐free survival for all children with DCM has improved over the last two decades, likely due to improved medical treatment of heart failure.^13^ This includes growth in the number of pediatric cardiologists with subspecialty expertise in cardiomyopathy and heart failure specific management^30^ and in the development of heart failure management guidance specific to pediatrics,^21^, ^22^, ^23^ including those with neurologic or muscular diseases, such as Barth syndrome.^28^ Additional steps needed to improve Barth syndrome cardiac care will include focused study of the mechanisms of cardiac involvement^31^, ^32^ and potential disease specific therapies.^33^ Furthermore, we strongly advocate for lifelong cardiac monitoring of all individuals with Barth syndrome, including those who demonstrate extended recovery. We make this recommendation given that: 1) relatively little is known about specific triggers for cardiac dysfunction in Barth syndrome, 2) late childhood onset cardiac dysfunction with severe heart failure with evidence of prior normal echocardiography has been reported in Barth syndrome,^6^ and 3) the relationship of arrhythmic sudden death with ventricular function/heart failure in Barth syndrome is not currently understood but potentially widely impactful for individuals with Barth syndrome.

In summary, we report on five children with Barth syndrome who presented symptomatic heart failure and LV dilation and systolic dysfunction in early life who achieved long‐term improvements of heart failure symptoms and near normalization to normalization of LV size and systolic function with timely diagnosis and medical heart failure management.

## CONFLICT OF INTEREST

Dr Feingold serves voluntarily on the Barth Syndrome Foundation Scientific and Medical Advisory Board. He also serves as a consultant for Stealth Biotherapeutics. Dr Yester has no relevant disclosures.

## AUTHOR CONTRIBUTIONS

Dr Yester performed chart review, assisted in conceptualization of the work, drafted the manuscript, figures, and table, and critically revised the manuscript. Dr Feingold conceptualized the work, guided data abstraction, and led critical revision of the manuscript, figures, and table.

## ETHICS STATEMENT

This research was performed by approval of the University of Pittsburgh Human Research Protection Office (PRO# 19050115) via waiver of informed consent.

## DISCLOSURES

Dr. Feingold serves as guarantor for the article, accepts full responsibility for the work and/or the conduct of the study, had access to the data, and controlled the decision to publish. This work was supported by the Thomas and Jennifer McCrady Heart Failure and Transplantation Research Fund at UPMC Children's Hospital of Pittsburgh.

## Data Availability

The data that support the findings of this study are not publicly available due to privacy or ethical restrictions.
